# Depositing Molecular Graphene Nanoribbons on Ag(111) by Electrospray Controlled Ion Beam Deposition: Self‐Assembly and On‐Surface Transformations

**DOI:** 10.1002/anie.202111816

**Published:** 2022-02-16

**Authors:** Wei Ran, Andreas Walz, Karolina Stoiber, Peter Knecht, Hongxiang Xu, Anthoula C. Papageorgiou, Annette Huettig, Diego Cortizo‐Lacalle, Juan P. Mora‐Fuentes, Aurelio Mateo‐Alonso, Hartmut Schlichting, Joachim Reichert, Johannes V. Barth

**Affiliations:** ^1^ Physics Department E20 Technical University of Munich James Franck Straße 1 85748 Garching Germany; ^2^ POLYMAT University of the Basque Country UPV/EHU Avenida de Tolosa 72 20018 Donostia-San Sebastian Spain; ^3^ Ikerbasque, Basque Foundation for Science Bilbao Spain

**Keywords:** Electrospray Deposition, Graphene Nanoribbons, Scanning Tunnelling Microscopy, Ultra-High Vacuum, On-Surface Synthesis

## Abstract

The chemical processing of low‐dimensional carbon nanostructures is crucial for their integration in future devices. Here we apply a new methodology in atomically precise engineering by combining multistep solution synthesis of N‐doped molecular graphene nanoribbons (GNRs) with mass‐selected ultra‐high vacuum electrospray controlled ion beam deposition on surfaces and real‐space visualisation by scanning tunnelling microscopy. We demonstrate how this method yields solely a controllable amount of single, otherwise unsublimable, GNRs of 2.9 nm length on a planar Ag(111) surface. This methodology allows for further processing by employing on‐surface synthesis protocols and exploiting the reactivity of the substrate. Following multiple chemical transformations, the GNRs provide reactive building blocks to form extended, metal–organic coordination polymers.

## Graphical abstract

N‐doped molecular graphene nanoribbons, deposited by electrospray controlled ion beam deposition, form extended, metal–organic coordination polymers after multiple chemical transformations employing on‐surface synthesis protocols. These transformations and the nature of the metal–organic coordination motifs are investigated by high‐resolution scanning tunnelling microscopy.

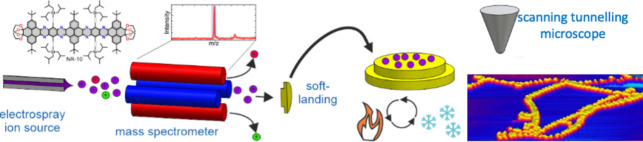

## Introduction

Graphene as well as other 2D sheet materials are extremely promising for innovative organic nano‐scale electronics. However, geometrically and chemically tailored derivatives are necessary to realize the envisioned circuits and materials to be employed in thermally and electrically conducting elements, sensors, transparent electrodes for displays, solar cells, catalysts, electrodes for batteries and fuel cells, and organic field‐effect transistors.^[1]^ These include graphene nanoribbons (GNRs): a class of materials whose electronic, optical and mechanical properties present significant application potential in electronics,[^1c^, ^2^] photonics,^[3]^ and energy storage^[4]^ and conversion.^[5]^ Structural modifications to control the semiconducting properties are achieved by limiting the dimensions to narrow strips, by designing edge structures, through doping the core or edges with heteroatoms, or by introducing heterojunctions.[^5a^, ^6^]

The physical properties of GNRs depend on the width, length, edge structure and doping loci, which makes synthesis with atomic precision crucial. Several top‐down strategies including slicing or etching of graphene, cutting, oxidation, intercalation, plasma etching of nanotubes and sonication of sheets frequently fail in this respect: the achieved geometries are too large, their nanostructures poorly defined, without atomic precision. Thus, alternative bottom‐up solution^[7]^ and on‐surface^[8]^ synthesis approaches were invoked, since they can provide control over all the structural parameters that determine the GNR properties. *On*‐*surface* synthesis typically requires a sublimation procedure for controlled surface deposition of thermally robust precursors under ultra‐high vacuum (UHV) conditions. Non‐UHV strategies to deposit the reactants on a given substrate, like spin coating, ink‐jet printing, drop‐casting, electrospray or chemical bath deposition typically do not meet the necessary purity requirements on an atomic scale and thus well‐defined molecular layers are elusive. By depositing tailored molecules on well‐defined interfaces in vacuum, novel, intricate structures and device elements come in reach. However, established deposition methodologies with atomic finesse (e.g. organic molecular beam epitaxy) are currently limited to thermostable sublimable molecules, whence a plethora of organic and almost all biomolecules with their fascinating properties are excluded.

Such limitations can be overcome by deposition systems using electrospray ionisation under UHV.^[9]^ This allows for ultra‐pure layers of soft‐landed and therefore integrity‐preserved species, a prerequisite for sophisticated high‐level nano‐scale material design and characterization.^[10]^ Literature provides many examples including biologically relevant molecules ((poly)saccharides,[^9b^, ^11^] peptides,^[12]^ proteins,^[13]^ DNA),[^9c^, ^14^] organic molecules,^[15]^ nano‐clusters,[^9a^, ^16^] and cage complexes.^[17]^ For this purpose and inspired by previous achievements, we have developed a system with innovative features, designated electrospray controlled ion beam deposition (ES‐CIBD), that processes readily dissolvable species including thermolabile and fragile entities like most organic and biological molecules.^[14]^ Our ES‐CIBD apparatus provides a preparative tool using digital ion‐guiding and mass‐spectrometry elements to transfer solvated molecules under ambient conditions onto well‐defined surfaces under UHV conditions with mass‐selectivity and tuneable energy.

**Figure 1:**
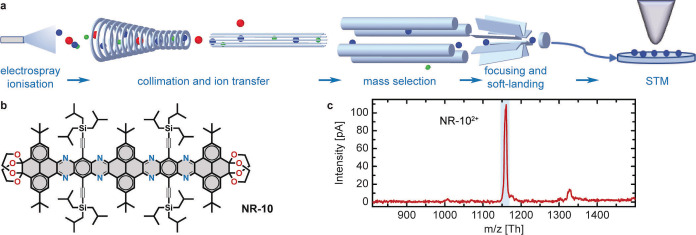
Electrospraying followed by controlled ion beam deposition of NR‐10. a) Ions produced via electrospray ionisation from a solution are transferred to ultra‐high vacuum by various ion guides in several pressure stages where neutral solvent molecules are removed. Ionic impurities are eliminated by a subsequent digital quadrupole mass spectrometer, which selects the target ions according to *m*/*z*. For soft‐landing, these ions are deposited on a surface with low kinetic energy (typically below 10 eV/*z*). The sample can be further investigated by a scanning tunnelling microscope. b) Chemical structure of NR‐10 (C_148_H_190_N_8_O_8_Si_4_). c) Mass spectrum of NR‐10 in THF solution. The main intensity at approximately 1162 Th originates from the doubly charged monomer. The ion beam was purified with the dQMS according to the deposition window marked by the shaded rectangle to exclusively deposit the NR‐10 attributed ions.

Herein, we demonstrate the clean deposition of complex, molecular GNRs, presenting a significant advancement beyond simple electrospray ionisation deposition of such graphene‐based macromolecules.^[18]^ Once on the surface, we further employ an on‐surface synthesis methodology,^[19]^ which may afford polymers via surface assisted reactions triggered by thermal annealing.^[20]^ The GNR species employed exhibits multiple functional groups, some of which are known to be quite labile (e.g. acetals and silyl, Figure 1b). These functional groups are introduced to fulfil several reactivity and solubility requirements for solution synthesis. Despite these moieties’ lability and the size of the GNR backbone, the entire species is depositable, without any evidence of hyperthermally induced mechanochemistry,^[21]^ giving rise to well‐ordered, self‐assembled structures on the Ag(111) surface as shown by scanning tunnelling microscopy (STM) investigations. Similarly to the protection and deprotection schemes used in solution synthesis, on the silver surface, the silyl solubilizing groups can be directly cleaved and the acetals can be transformed into the corresponding diones, enabling the formation of metal‐organic coordination polymers. The stepwise conversion and polymerisation scheme of the small GNR is characterised by analysis of high‐resolution STM images.

## Results and Discussion

We employed a molecular N‐doped conjugated polyaromatic species, which is obtained by multistep organic synthesis in solution.^[7i]^ The molecule under investigation is a linear nanoribbon with 10 conjugated linearly fused rings (NR‐10, Figure 1b), a molecular mass of 2321.54 Da (calculated from the isotopic composition and in full agreement with literature)^[7i]^ and a length of 2.9 nm.

The atomically clean deposition of non‐volatile molecules in UHV is performed by ES‐CIBD (schematic shown in Figure 1a).^[22]^ Starting from a sample solution with dissolved analyte molecules, a beam of gas‐phase ions is generated via electrospray ionisation. Transfer ion guides conduct the ion beam from the ambient conditions of the spray to UHV and a digital quadrupole mass spectrometer (dQMS). Here, the beam gets either analysed for its composition or filtered according to *m*/*z* for subsequent deposition. The dQMS exclusively transmits a small window of *m*/*z* values with adjustable width and position while all other ions are removed from the beam. Finally, soft‐landing of ions with low kinetic energies yields intact molecules on the sample surface, where the coverage is controlled by the incident ion beam intensity. The combination with a variable temperature UHV‐STM and an in situ transfer system allows for investigation and further manipulation of the deposited layers. The subsequent STM characterisation showed that high‐purity depositions were achieved.

Positive‐mode electrospray of the NR‐10 molecules dissolved in an acidified THF/water mixture (cf. Methods) resulted in the mass spectrum depicted in Figure 1c. The doubly charged monomer at around 1162 Th is the dominant ion species and therefore was selected for all depositions. Soft‐landing was performed with ≈2 eV/*z* kinetic energy per molecule (≈4 eV at *z*=2) on the Ag(111) surface held at room temperature.

**Figure 2:**
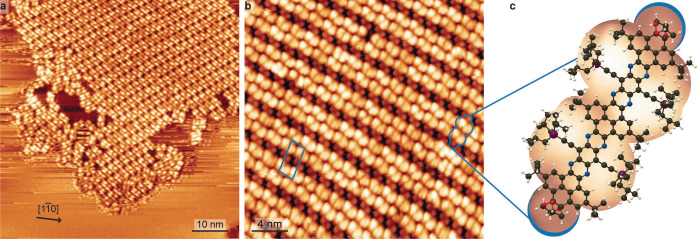
STM imaging of NR‐10 on Ag(111). a) Surface overview in long range micrograph (111 K, −1.76 V, 0.15 nA). b) Magnified view of self‐assembled island (160 K, 2.1 V, 0.12 nA). c) Cropped image of identified single molecule, overlaid with NR‐10 molecular model. C, N, O, Si and H are depicted in black, blue, red, purple and white, respectively.

The samples were subsequently examined by STM. Annealing to temperatures up to 423 K affected neither the single molecule appearance nor the long‐range order. Exemplary data are shown in Figure 2. In general, on atomically planar Ag(111) terraces we found molecular islands surrounded by diffusing molecules (see high contrast and streaking observed outside the molecular island) as shown in Figure 2a. Zooming in such an island allows to identify the self‐assembly as a regular structure which can be described by the epitaxial matrix $\left(\begin{array}{cc} 10 & 15\\ 4 & -3 \end{array}\right)$ (Figure 2b). This arrangement is presumably mediated by van‐der‐Waals interactions between the tri‐isobutylsilyl (TIBS) substituents. Different packing geometries were found (see Supporting Information Figure S1), a further testament to the lack of a strong molecular registry to the Ag(111) as well as directorial intermolecular interactions. Within the self‐assembled structures, we can identify the single molecules (Figure 2c). Their contrast is dominated by the topographically protruding moieties. Brighter, asymmetric protrusions signify the bulky TIBS groups. Two smaller, round protrusions mark the positions of the terminal acetals.

We proceeded to investigate systematically the on‐surface thermal chemistry and polymerisation of these well‐defined nanoribbons by annealing in steps of 20 K followed by STM examination (see Supporting Information Figure S2 for a compilation of STM images of all the annealing steps).

**Figure 3:**
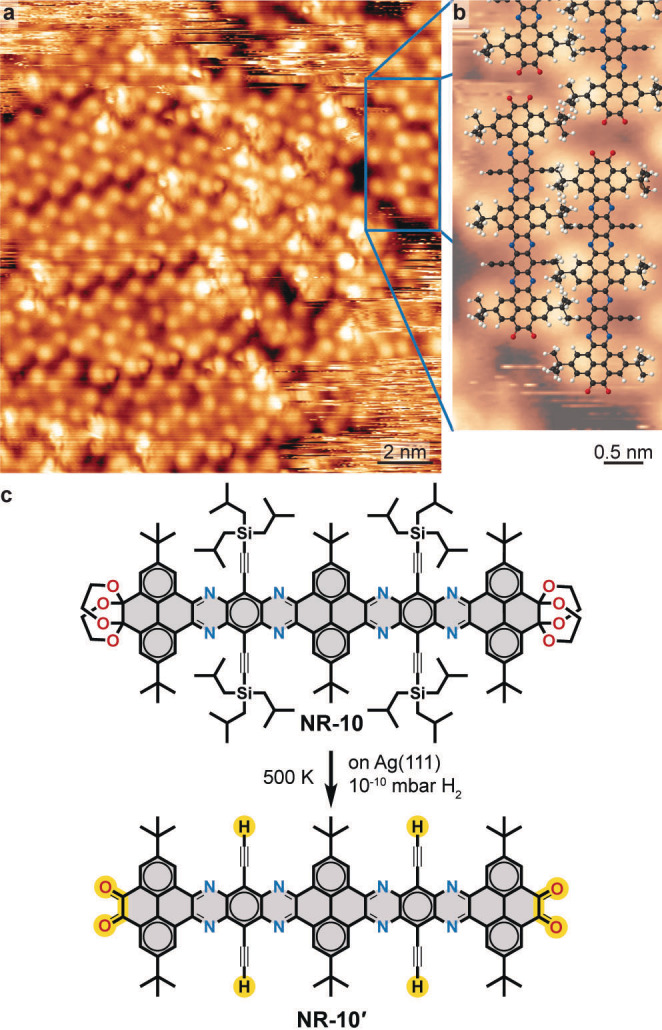
On‐surface double deprotection of NR‐10. a) Overview STM image (130 K, 1.25 V, 0.09 nA) of NR‐10 on Ag(111) after annealing at 503 K. b) Magnification of the area indicated in (a) overlaid with proposed molecular model of NR‐10′. C, N, O and H are depicted in black, blue, red and white, respectively. c) Scheme of proposed on‐surface NR‐10 to NR‐10′ transformation.

After annealing at 483 K, we observed that some NR‐10 molecules loose the brighter protrusions corresponding to the TIBS groups and almost all of them were removed by annealing at 503 K (Figure 3a, b). The nanoribbon is now characterised by three pairs of less bright protrusions corresponding to the tertiary butyl (^*t*^Bu) substituents and a faint appearence of the GNR backbone.^[23]^ This is attributed to on‐surface deprotection of the TIBS‐terminated alkyne moieties, analogous to the deprotection of trimethylsilyl terminated alkynes on the same surface.^[24]^ The cleaved TIBS groups were not identified in our STM images. The TIBS deprotection can lead to diacetylene linked monomers or to surface‐stabilised radicals.[^24^, ^25^] As the TIBS deprotected NR‐10 molecules showed no evidence of a reactive alkynyl group, and given the small residual pressure of H_2_ gas (≈1×10^−10^ mbar) in the UHV environment, we propose a H passivation of the alkynyl group.^[26]^ The TIBS deprotected NR‐10 seem to be interacting by van der Waals forces of interdigitated ^*t*^Bu groups. Outside the self‐assembled island, one can identify streaking, presumably caused by mobile adsorbates. These may be attributed to partially TIBS deprotected NR‐10 for which the interdigitation of ^*t*^Bu groups, and hence their immobilization in the two‐dimensional structure, cannot be accommodated.

We further notice the lack of topographic features in the positions of the terminal groups, which, based on the STM contrast,^[23]^ indicates their deprotection.^[7i]^ Therefore we propose the chemical transformation of NR‐10 to the doubly deprotected NR‐10′ (Figure 3c).

**Figure 4:**
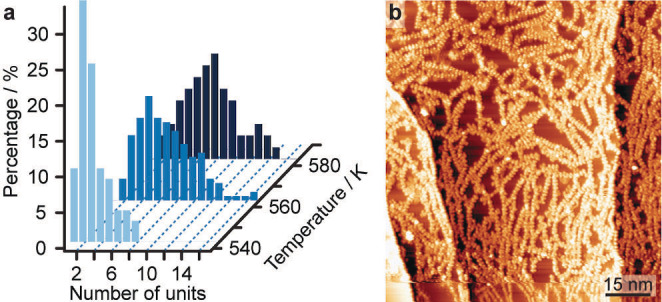
On‐surface polymerisation of NR‐10′. a) Distributions of polymerized GNR length as a function of temperature. b) Overview STM image (159 K, 1.7 V, 0.11 nA) after annealing to 583 K.

**Figure 5:**
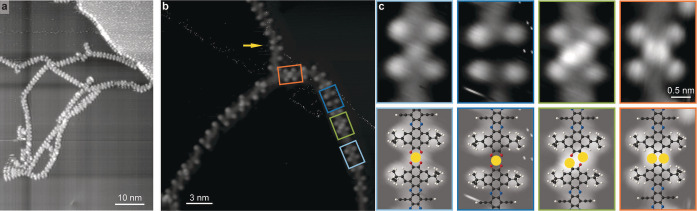
Polymer node identification. a) STM image showing robust chain binding with different node flexibility (167 K, 1.8 V, 0.15 nA). b) High‐resolution STM image displaying four different types of dimer nodes (123 K, 1.5 V, 0.07 nA). c) Magnification of the nodes identified in the STM image in (b) directly compared with proposed molecular model. Ag, C, N, O and H are depicted in yellow, black, blue, red and white, respectively.

In the subsequent annealing step at 523 K, we observe the onset of an on‐surface extension of NR‐10′ by linking at the terminal positions of the nanoribbon. The polymerisation is promoted by increasing annealing temperatures (Figure 4a), whereby eventually the surface is covered by molecular chains (Figure 4b). Along with the polymerisation, we also observe the onset of decomposition at the ^*t*^Bu groups, as judged by occasionally diminished contrast at the related positions. As a uniform contrast of the ^*t*^Bu groups is found in the STM images after annealing at 503 K (see Figure 3) and ^*t*^Bu group defects occur more frequently after annealing at 573 K (see Figure 5b), this decomposition can be attributed to the higher temperature annealing, in good accord with our earlier report for a shorter tetraketone monomer.^[27]^

To identify the linking mode within the chains, we prepared a surface of lower molecular coverage (Figure 5a). Here we could clearly find chains constructed of single molecules, occasionally interlinking with segments that pivot at particular nodes. Thus, we can deduce the following: 1) the polymer links are not uniform across the NR‐10′ polymer and 2) the chain is robustly linked. The links have been analysed as detailed in the Supporting Information Figures S3, S4. Among the links we can identify previously reported nodes of Ag‐O_4_, forming with the native Ag adatoms (indicated by blue frames).^[27]^ The different apparent height of the metal node might originate from a variation in the adsorption site, accommodation of more than a single adatom or ligation of small adducts (due to residual gases in the vacuum or molecular fragments related to the monomer's chemical modification). Their formation is linked to the hybridisation of the terminal ketone groups with the silver and their conversion to catecholates. In addition, we can recognise nodes with paired bright protrusions (green and orange frames). Their imaging being in good accord with reports of Ag atoms in planar organometallic complexes,^[28]^ we tentatively attribute these to Ag adatoms. Using the determined positions of the ^*t*^Bu substituents to evaluate the distance between the monomers (Figure S3), we find that the assigned Ag adatoms are located at positions that cannot accommodate the terminal O atoms in a planar geometry (as illustrated in Figure S3f). We therefore propose that these species are directly linked to the terminal C atoms of the nanoribbon monomers. Hence we derive a model that features the consecutive expression of links with double bridges of either C−Ag−O−C (framed in green in Figure 5b, c) or direct C−Ag−C (framed in orange in Figure 5b, c). Intermediate steps in the linking may result in a tilted geometry between the monomers (example indicated by arrow in Figure 5b). Here, without the clear signature of the position of an Ag adatom, it is not possible to propose an exact bonding motive (see Supporting Information Figure S4).

## Conclusion

We have demonstrated that ES‐CIBD is a promising method for controlling the deposition and processing of thermolabile nanographenes. The exquisite control over the process allows for high‐quality depositions with small amounts of analyte of a bulky N‐doped GNR. The well‐defined nature of the deposited species is characterized by STM, revealing a long‐range interfacial ordering on the Ag(111) surface. Accordingly, such preparations are suitable for fabricating samples to be further scrutinized by space‐averaging analysis techniques towards obtaining complete information about the physicochemical properties of the surface‐confined GNRs. Moreover, temperature‐induced transformations of N‐doped GNR afforded metal–organic polymers of up to 50 nm length. These are assigned to a variation of metal–organic and organometallic nodes incorporating native Ag adatoms, based on high‐resolution STM insights. Thus, ES‐CIBD is anticipated to facilitate further novel schemes of on‐surface synthesis and integration of nanographenes or nano‐objects in functional architectures at interfaces.

## Conflict of interest

The authors declare no conflict of interests.

## Supporting information

As a service to our authors and readers, this journal provides supporting information supplied by the authors. Such materials are peer reviewed and may be re‐organized for online delivery, but are not copy‐edited or typeset. Technical support issues arising from supporting information (other than missing files) should be addressed to the authors.

Supporting Information
