# Supplementary material for: Depositing Molecular Graphene Nanoribbons on Ag(111) by Electrospray Controlled Ion Beam Deposition: Self‐Assembly and On‐Surface Transformations
Source: Angew Chem Int Ed Engl. 2022 Feb 16;61(14):e202111816. doi: 10.1002/anie.202111816 (PMC9305426; doi:10.1002/anie.202111816)
Supplement: Supplementary file 1 — Supporting Information [file ANIE-61-0-s001.pdf]

Angewandte Chemie
Eine Zeitschrift der Gesellschaft Deutscher Chemiker

Supporting Information

Depositing Molecular Graphene Nanoribbons on Ag(111) by Electrospray Controlled Ion Beam Deposition: Self-Assembly and On-Surface Transformations

W. Ran, A. Walz, K. Stoiber, P. Knecht, H. Xu, A. C. Papageorgiou, A. Huettig, D. Cortizo-Lacalle, J. P. Mora-Fuentes, A. Mateo-Alonso, H. Schlichting, J. Reichert*, J. V. Barth**

Supporting Information
©Wiley-VCH 2021
69451 Weinheim, Germany

Depositing Molecular Graphene Nanoribbons on Ag(111) by Electrospray Controlled Ion Beam Deposition: Self-assembly and On-Surface Transformations

Wei Ran, Andreas Walz, Karolina Stoiber, Peter Knecht, Hongxiang Xu, Anthoula C. Papageorgiou,* Annette Huettig, Diego Cortizo-Lacalle, Juan P. Mora-Fuentes, Aurelio Mateo-Alonso, Hartmut Schlichting, Joachim Reichert,* and Johannes V. Barth *

Table of Contents

Experimental Procedures 2
Sample preparation 2
ES-CIBD 2
Results and Discussion 3
Figure S1: STM images of three different self-assembled phases of NR-10 on Ag(111) 3
Figure S2: NR-10 on Ag(111) assembly and chemical reactivity by thermal annealing. 4
Figure S3: Details of polymer node identification 4
Figure S4: Details of ambiguous polymer node 5
References 5

Experimental Procedures

Sample preparation

The samples were prepared in a UHV set-up (base pressure of 2×10^{-10} mbar) connecting the ES-CIBD and the STM apparatus.[1] Prior to the ES-CIBD the atomically planar surface of Ag(111) (Surface preparation laboratory, single crystal polished to ~0.1°) was cleaned by multiple cycles of Ar^+ sputtering (1 kV) and UHV annealing (700 K) and checked by STM.

ES-CIBD

NR-10 was synthesised according to previously reported procedures.[2] Detailed deposition procedures with the ES-CIBD system are described elsewhere.[3] For the experiments here, a solution of 3×10^{-5} M NR-10 dissolved in a THF water mixture (94 % / 6 % v/v) and 2×10^{-3} M trichloroacetic acid was used. The respective mass spectrum with the dominant ionic species at ~ 1162 Th reflects the doubly charged monomer (Figure 1c). Prior to deposition in UHV, the ion beam was purified with the dQMS with a resolution of about 70, cutting off the small peak about 15 Th above the NR-10 attributed peak. The highly aprotic composition of the predominantly organic solvent required for this kind of molecule, led to a low conductivity of the electrolyte, which resulted in a rather low efficiency of ionization. The emitter voltage was 3.5 kV, the flow rate 60-90 µlh^{-1}, the ion current up to 100 pA, the deposition time approximately 1.5 hours, the base pressure $<5\times10^{-10}$ mbar and soft-landing was performed with a kinetic energy of 2 eV/z.

STM

STM measurements were conducted with a commercial Aarhus 150 STM (SPECS GmBH). Data were recorded by electrochemically etched W tips with the tunnelling bias applied to the sample. The images were processed (planarization and contrast adjustment) with the WSxM[4] software package.

Results and Discussion

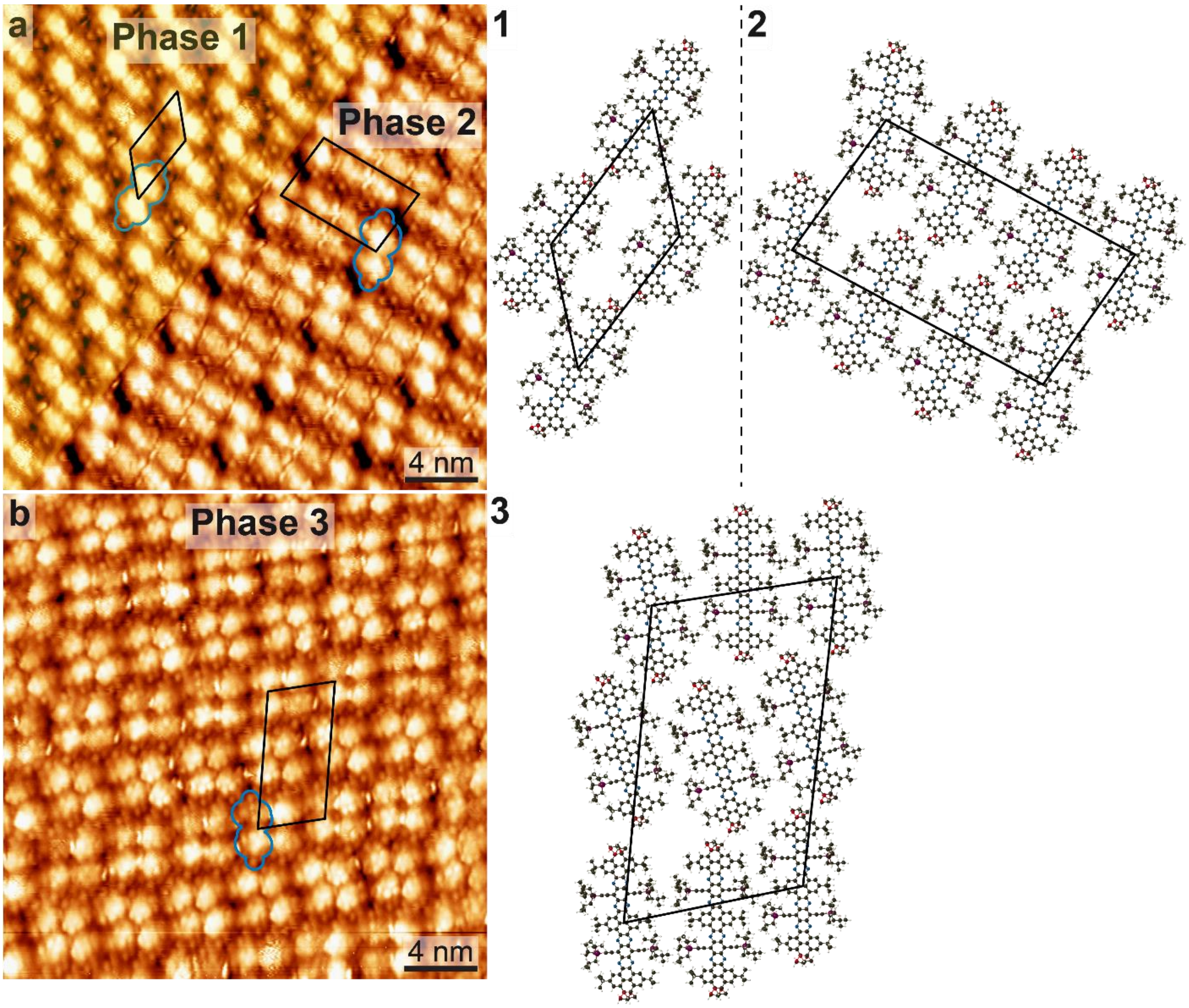

Figure S1. STM images of three different self-assembled phases of NR-10 on Ag(111) (a: 153 K, 2.1 V, 0.14 nA, b: 170 K, 2.1 V, 0.14 nA). A single intact molecule is outlined in blue in each phase. The unit cells are indicated in the images. Structural models of the phases 1, 2 and 3 are shown to the right of the respective STM images.

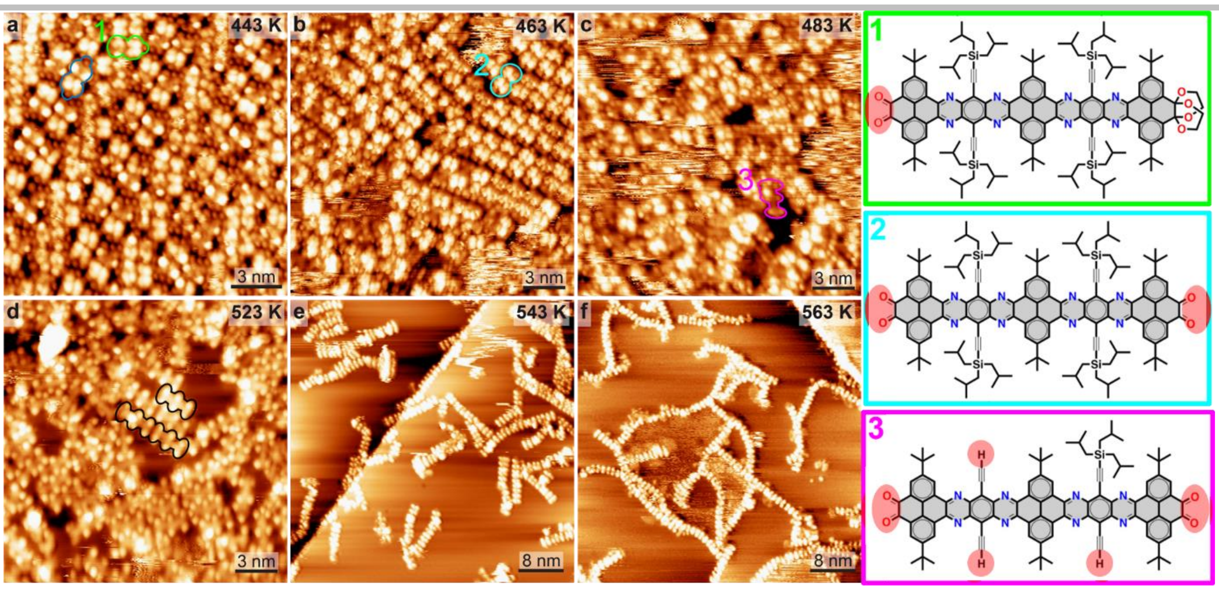

Figure S2. NR-10 on Ag(111) assembly and chemical reactivity by thermal annealing. (a-f) STM images of NR-10 and its reaction products after annealing to 443 K, 463 K, 483 K, 523 K, 543 K, 563 K, respectively. One single intact molecule is outlined in blue in image a. The reaction products after annealing at different temperatures are outlined in different colours in images a, b and c and labelled with 1, 2 and 3, respectively. The chemical structures of 1, 2 and 3 are displayed beside STM images (chemical changes highlighted in red). (a: 143 K, 2.1 V, 0.11 nA; b: 142 K, 2.1 V, 0.13 nA; c: 120 K, 2.1 V, 0.13 nA; d: 142 K, 1.9 V, 0.10 nA; e: 140 K, 0.8 V, 0.06 nA; f: 162 K, 2.2 V, 0.09 nA)

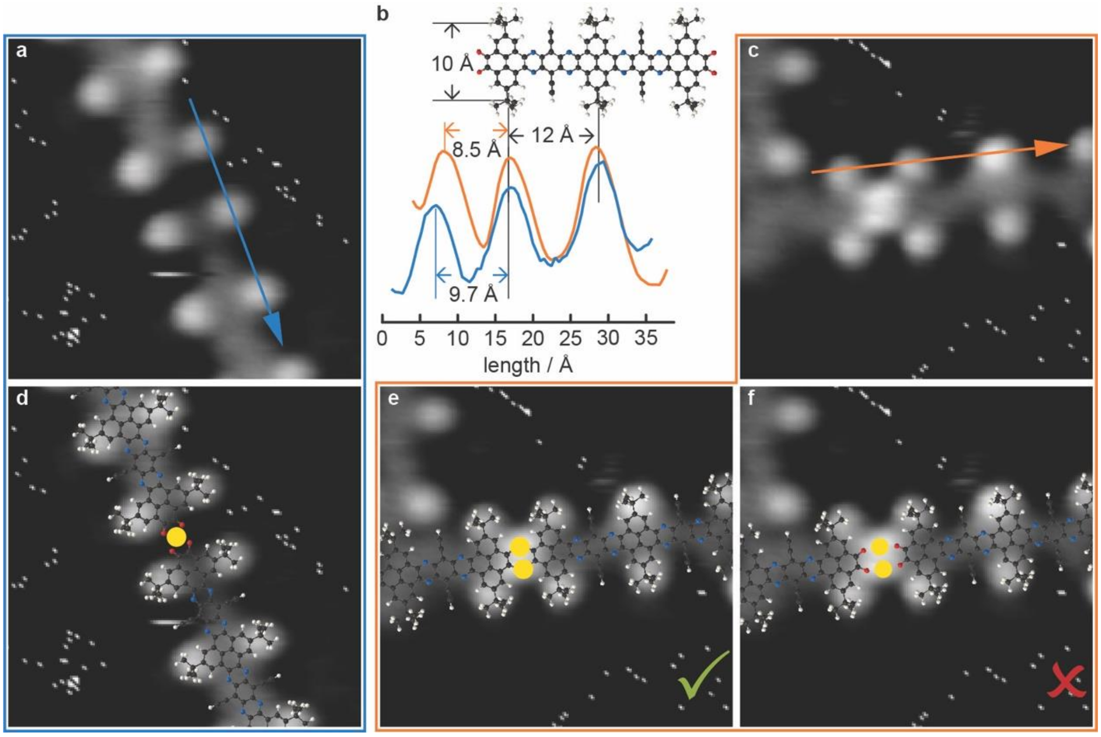

Figure S3. STM images with NR-10′ polymers and node assignment. (b) A molecular model of an isolated NR-10′ molecule with the indicated distances of the ^{t}Bu groups obtained from the optimised geometry (B3LYP 6-31g) of NR-10[2] is used for the image calibration. C, O, N and H are depicted in black, red, blue and white, respectively. Line profiles along two different nodes (shown in the STM images in (a) and (c)) indicate the fit, which uses the peak maxima. The distances measured between the monomer's ^{t}Bu groups have an accuracy of ~ 0.5 Å in the globally calibrated image (complete image shown in Figure 5b). This results in an accuracy of the measurement of the distance between the closest ^{t}Bu groups of neighbouring monomers of ~ 1 Å. This accuracy is very close to the expected separation variations resulting from the different proposed node structures, however small differences are detectable, in line with the proposed models. (d,e) Proposed models for the nodes. The additional protrusions at the nodes are associated with Ag metal adatoms (yellow circles) native on the Ag(111). The bond distances in the proposed models are consistent with literature values.[5] The assignment of the bright protrusions to Ag adatoms on the node shown in c, e, f (outlined in orange in Figure 5c) cannot be substantiated with the presence of the monomer's O atoms in a planar geometry: with a Ag–O bond of ~ 2.3 Å[6] and a Ag–C bond of ~ 2.1 Å[7] this would require an estimated extra 2 Å space between the closest ^{t}Bu groups of neighbouring molecules. The model in (f), which presents the possibility of two paired Ag–O bonds with a length of 2.3 Å, cannot be reconciled with the STM topographic data.

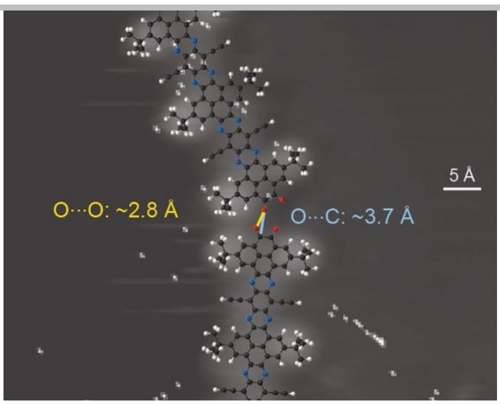

Figure S4. Zoom in STM image showing a kinked node overlaid with monomer models. The closest projected O···O distance in the model (~ 2.8 Å) might indicate that a C–O bond is broken and a C–Ag–O–C link has formed. Alternative plausible coordination schemes include the incorporation of a three-fold O coordinated Ag adatom.

References

[1] A. Walz, Doctoral thesis, Technische Universität München (München), **2020**.
[2] D. Cortizo-Lacalle, J. P. Mora-Fuentes, K. Strutyński, A. Saeki, M. Melle-Franco, A. Mateo-Alonso, *Angew. Chem. Int. Ed.* **2018**, *57*, 703-708.
[3] A. Walz, K. Stoiber, A. Huettig, H. Schlichting, J. V. Barth, **2021**, DOI: 10.33774/chemrxiv-32021-gw33753x-v33772.
[4] I. Horcas, R. Fernández, J. M. Gómez-Rodríguez, J. Colchero, J. Gómez-Herrero, A. M. Baro, *Rev. Sci. Instrum.* **2007**, *78*, 013705.
[5] a) A. C. Papageorgiou, J. Li, S. C. Oh, B. Zhang, Ö. Sağlam, Y. Guo, J. Reichert, A. B. Marco, D. Cortizo-Lacalle, A. Mateo-Alonso, J. V. Barth, *Nanoscale* **2018**, *10*, 9561-9568; b) S. Kawai, A. Sadeghi, T. Okamoto, C. Mitsui, R. Pawlak, T. Meier, J. Takeya, S. Goedecker, E. Meyer, *Small* **2016**, *12*, 5303-5311.
[6] E. M. Njogu, B. Omondi, V. O. Nyamori, *J. Coord. Chem.* **2017**, *70*, 2796-2814.
[7] U. Hintermair, U. Englert, W. Leitner, *Organometallics* **2011**, *30*, 3726-3731.
